# Corrigendum

**DOI:** 10.1002/mpr.1921

**Published:** 2022-12-04

In the article titled ‘The effect of early cognitive training and rehabilitation for patients with cognitive dysfunction in stroke’ (Xuefang et al., 2021), the corresponding author's mailing address should be changed to:

Department of Neurology, Aerospace Center Hospital, No. 15 Yuquan Road, Haidian District, Beijing

The Ethics Approval and Consent to Participate section should be updated to:

Ethical approval was obtained from IEC of Aerospace Center Hospital. Informed consent was obtained from the participants with the option to withdraw them from the study at any time. Consent for publication IEC, Department of Neurology, Aerospace Center Hospital approved the publication of data generated from this study.
